# Impact of the Graphite Fillers on the Thermal Processing of Graphite/Poly(lactic acid) Composites

**DOI:** 10.3390/ma14185346

**Published:** 2021-09-16

**Authors:** Daniel Kaczor, Kacper Fiedurek, Krzysztof Bajer, Aneta Raszkowska-Kaczor, Grzegorz Domek, Marek Macko, Piotr Madajski, Pawel Szroeder

**Affiliations:** 1 Faculty of Mechatronics, Kazimierz Wielki University, Kopernika 1, 85-074 Bydgoszcz, Poland; kacper.fiedurek@impib.lukasiewicz.gov.pl (K.F.); gdomek@ukw.edu.pl (G.D.); mackomar@ukw.edu.pl (M.M.); 2 Łukasiewicz Research Network-Institute for Engineering of Polymer Materials and Dyes, Marii Skłodowskiej-Curie 55, 87-100 Toruń, Poland; krzysztof.bajer@impib.lukasiewicz.gov.pl (K.B.); aneta.kaczor@impib.lukasiewicz.gov.pl (A.R.-K.); 3 Faculty of Chemistry, Nicolaus Copernicus University, Gagarina 7, 87-100 Toruń, Poland; piotr.madajski@doktorant.umk.pl; 4 Institute of Physics, Kazimierz Wielki University, Powstańców Wielkopolskich 2, 85-090 Bydgoszcz, Poland; psz@ukw.edu.pl

**Keywords:** poly(lactic acid), graphite, composites, room temperature crystallinity, oxidation induction time, melt flow rate

## Abstract

To assess the impact of graphite fillers on the thermal processing of graphite/poly(lactic acid) (PLA) composites, a series of the composite samples with different graphite of industrial grade as fillers was prepared by melt mixing. The average size of the graphite grains ranged between 100 µm and 6 µm. For comparative purposes, one of the carbon fillers was expandable graphite. Composites were examined by SEM, FTIR, and Raman spectroscopy. As revealed by thermogravimetric (TG) analyses, graphite filler slightly lowered the temperature of thermal decomposition of the PLA matrix. Differential scanning calorimetry (DSC) tests showed that the room temperature crystallinity of the polymer matrix is strongly affected by the graphite filler. The crystallinity of the composites determined from the second heating cycle reached values close to 50%, while these values are close to zero for the neat polymer. The addition of graphite to PLA caused a slight reduction in the oxidation induction time (OIT). The melt flow rate (MFR) of the graphite/PLA composites was lower than the original PLA due to an increase in flow resistance associated with the high crystallinity of the polymer matrix. Expandable graphite did not cause changes in the structure of the polymer matrix during thermal treatment. The crystallinity of the composite with this filler did not increase after first heating and was close to the neat PLA MFR value, which was extremely high due to the low crystallinity of the PLA matrix and delamination of the filler at elevated temperature.

## 1. Introduction

Poly(lactic acid) produced from renewable resources has currently a principal position on the market of biodegradable polymers [1]. To tailor its properties to specific engineering applications, such as mechanical and automotive parts, electronic and electrical devices, or electrodes in electrochemical devices, it is necessary to tune its properties by combining the polymer matrix with different dispersed phases [2]. Carbon nanomaterials such as carbon nanotubes and graphene with its superior thermal and electrical properties can be used as a filler that improves some specific properties, such as stiffness, thermal stability, fire retardancy, and lower permeability [3]. The addition of graphene oxide to the polymer matrix results in the improvement of the Young modulus and an inhibition of the bacteria proliferation [4]. Due to the improved mechanical properties and electrical conductivity, graphene/PLA composites are considered as a filament for 3D printing [5] and an efficient electromagnetic interference shielding material [6,7].

Most pristine PLAs are in an amorphous state and tend to degrade during thermal processing, presenting a rapid reduction of molecular weight due to a polymer chain scission [8,9]. Graphene and graphite fillers do not only modify the properties of composites, but also affect the processability of the PLA matrix. The polymer crystallinity plays an important role in determining the physical properties that can be employed for specific applications of PLA during the processing, and it can be increased by nucleating agents. It has been shown that the crystallization behavior of PLA can be altered by the incorporation of the graphene oxides [10] and carbon nanotubes [11,12] into the PLA matrix. Use of the graphene oxide filler resulted in an increase of the degree of crystallinity to the level of 27% at cooling rate of 10 °C/min [10]. In turn, the crystallinity of the PLA matrix after the introduction of carbon nanotubes reaches the value of 20% [12]. As we show in this work, graphite fillers cause an increase in crystallinity above 40%.

The crystallization process assisted by the carbon fillers is influenced by the thermal history of the composite. In order to fully exploit the application potential of composites, apart from case studies on the specific functionalities of these materials, studies of the influence of industrial-grade graphite fillers on the processability of graphene and graphite/PLA are required.

To meet these needs, we conducted extensive studies of neat PLA (as reference) and PLA composites with various graphite fillers of industrial grade using FTIR and Raman spectroscopy, TGA, DSC, OIT, and MFR. As fillers, we used graphite powders of various grain sizes ranging from 6 to 100 µm and expandable graphite. The main focus of this work is the relationship between the graphite filler morphology and thermal phase transitions of the polymer matrix (glass transition, crystallization, and melting) that usually occur during the thermal processing. We show that industrial grade graphite powders can act as a nucleation agent and influence the polymer processing history by slowing down the degradation processes of the polymer matrix.

## 2. Materials and Methods

### 2.1. Materials

As a PLA matrix, the Ingeo™ Biopolymer 3260HP was selected, with density = 1.24 g/cm^3^, mass-average molar mass = 107.5 Da, and polydispersity index = 1.68, supplied by NatureWorks (Minnetonka, MN, USA).

The following graphite micropowders of industrial grade were used as fillers: MG192 (100 mesh, carbon content 92%), MG394 (325 mesh, carbon content 94%), MG1596 (1500 mesh, carbon content 96%), and MG3096 (3000 mesh, carbon content 96%). In addition to chemically unmodified powders, expandable graphite EG290 (+400 mesh, carbon content 80%, expansion temperature >200 °C) intercalated with sulphur compounds was also used. All carbon powders were supplied by Sinograf SA (Toruń, Poland).

Apart from drying, all materials were used as received.

### 2.2. Composite Preparation

Prior to the compounding, unprocessed PLA granulate was dried at a temperature of 80 °C for 8 h. Before introducing graphite fillers, the neat PLA resin was premixed at 190 °C for 2.5 min using a Plasti-Corder^®^ Lab-Station (Brabender, South Hackensack, NJ, USA). Graphite powders were added with an approximate weight ratio of 1:3 for a mixing time of 2 min, with a blade rate of 50 rpm, at 190 °C. The obtained blends were coded according to the type of graphite in the composite, as shown in Table 1.

### 2.3. Material Characterization

#### 2.3.1. Phase Morphology Analysis

Scanning electron microscopy (SEM, SU8010, Hitachi, Japan) was used for studying the morphology of both the graphite fillers and graphite/PLA composites. For SEM imaging, graphite flakes and micropowders were deposited on conductive carbon adhesive tapes.

#### 2.3.2. Chemical Structure Analysis

ATR-FTIR spectra were measured using the Agilent Technologies Cary 630 ATR-FTIR spectrometer (Agilent, Santa Clara, CA, USA), over the wavenumber range of 4000 cm^−1^ to 400 cm^−1^ with a resolution of 2 cm^−1^.

Raman spectra were recorded in backscattering geometry, with a Senterra Raman microscope (Bruker Optik, Billerica, MA, USA), using a 2 mW laser beam with a wavelength of 532 nm as an excitation light source. Both the ATR-FTIR and Raman spectra were acquired at ambient temperatures.

#### 2.3.3. Thermal Behavior and Stability Analysis

Thermal gravimetric analyses (TGA) were carried out using an analyzer TG/DSC STA 449 F5 Jupiter (NETZSCH Instruments, Selb, Germany). Samples were measured in a crucible with a mass of about 4–6 mg. Composites were tested under nitrogen at temperatures ranging from 30 °C to 1050 °C with a heating rate of 10 °C/min.

Differential scanning calorimetry (DSC) was performed with a calorimeter DSC1 (Mettler-Toledo, Swiss) calibrated with pure indium and zinc standards under nitrogen atmosphere at gas flow rate 50 cm^3^/min. Each sample of 5–7 mg was sealed in an aluminum crucible and heated from 0 °C to 300 °C. After the first heating scan, the sample in the crucible was cooled to 0 °C and reheated to 300 °C (second heating scan). The heating/cooling rate was 10 °C/min. Before the cooling and second heating scan, the sample was kept at a constant temperature of 300 °C and 0 °C for 3 min, respectively. The inflection point of each glass transition was taken as a glass transition temperature, $T_{g}$. Crystallization and melting peak position were taken as a crystallization $T_{c}$ and melting temperatures $T_{m}$, respectively. Crystallization and melting enthalpies were evaluated from the integrated areas of the peaks.

The room temperature crystallinity, *X_c_*, of PLA composites was evaluated using the following expression:

$$X_{c}=\left(\frac{\Delta H_{m}-\Delta H_{cc}}{w\Delta H_{m}^{0}}\right)\cdot 100\%$$

where Δ*H_m_* is the enthalpy of melting (J/g), Δ*H_cc_* is the cold crystallization enthalpy (J/g), $\Delta H_{m}^{0}$ is the melting enthalpy of 100% crystalline PLA (93 J/g), and *w* is the fraction of the polymer in the composite materials [13]. The experiment was performed in accordance with the ISO 11357-(1-3): 2009 standards.

The Mettler Toledo DSC1 calorimeter was also used to study thermal degradation of polymer matrix. OIT was measured at the isothermal temperature of 230 °C and gas flow rate of 50 cm^3^/min. Each sample of 6–8 mg was heated under nitrogen from 50 to 230 °C at heating rate of 10 °C/min. After reaching the required temperature, the sample was kept under nitrogen for 5 min to obtain thermodynamic equilibrium conditions. Then, the gas flow was changed from nitrogen to oxygen and, still under isothermal conditions, the onset of the exothermal signal (oxidation) was measured. The experiment was performed in accordance with the requirements of the ISO 11357-6:2018 standard.

The melt flow rate (MFR) of the composites was determined according to the PN-EN ISO 1133:2011 standard with a Dynisco LMI 4003 capillary plastometer. The measurements were carried out under the piston loading of 2.16 kg at 190 °C.

## 3. Results

### 3.1. Phase Morphology Analysis

**Figure 1:**
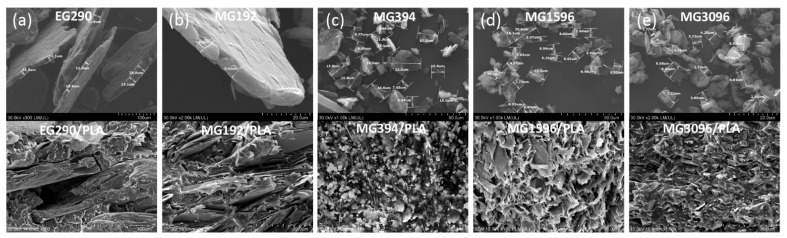
SEM images of neat graphite fillers (top) and graphite/PLA composites after mixing at 190 °C (bottom): (**a**) EG290/PLA, (**b**) MG192/PLA, (**c**) MG394/PLA, (**d**) MG1596/PLA and (**e**) MG3096/PLA.

Figure 1 shows SEM images of graphite fillers and graphite/PLA composites after melt mixing at 190 °C. In sample EG290, the size of the graphite flakes was found to be 400 ± 100 µm, while the thickness was about 20 µm. The mean sizes of flakes in MG192 was estimated to be 107 ± 55 µm and thickness was found to be about 10 µm. The average size of the MG394, MG1596, and MG3096 micropowders are 14 ± 5 µm, 10 ± 4 µm, and 6 ± 2 µm, respectively.

The morphology of the EG290/PLA composite (Figure 1a) differs significantly from other composites. We can see that the expandable graphite flakes EG290 delaminated during mixing. The structure of composites with graphite micropowders MG192, MG396, MG1596, and MG3096 is more homogeneous with the increasingly smaller size of the graphite grains (Figure 1b–e).

### 3.2. FTIR and Raman Analyses

**Figure 2:**
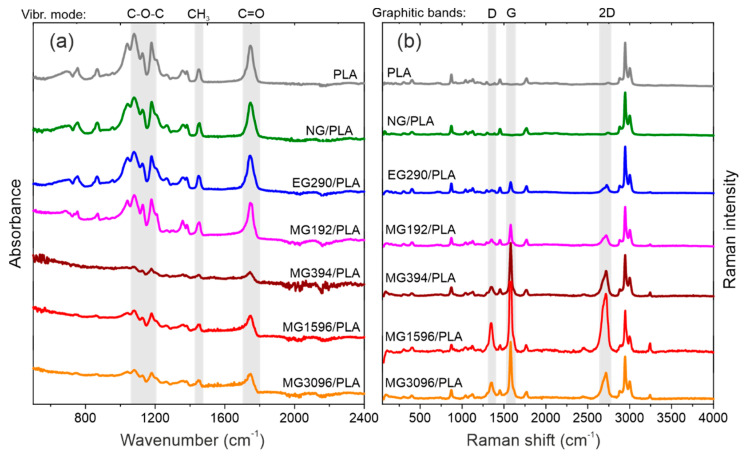
ATR-FTIR (**a**) and Raman scattering (**b**) spectra of graphite/PLA composites.

Figure 2 shows ATR-FTIR and Raman spectra of the composites. The upper spectra were obtained for the raw PLA and PLA subjected to the melting process at 190 °C (NG/PLA). The remaining spectra were obtained for samples of composites with graphite filler.

The infrared absorption spectra graphite/PLA composites contain the bands that are assigned to the PLA-based polymer [14,15]. Two bands at 755 and 868 cm^−1^ are attributed to the crystalline and amorphous phases of the polymer. The stretching modes of the C-CH_3_ group appear at 1041 cm^−1^ while the symmetric and asymmetric stretching modes of the C-O-C group appear at 1079 (symm.), 1180, and 1266 cm^−1^ (asymm.), respectively. The band at 1127 cm^−1^ is assigned to the rocking modes of the CH_3_ group. Characteristics of the CH and CH_3_ symmetric bending modes appear at 1360 and 1382 cm^−1^ while the band corresponding to the asymmetric bending modes is found at 1454 cm^−1^. Then, the C=O stretching modes of the ester group appear at 1746 cm^−1^. There are also weak bands at 2946 and 2994 cm^−1^ attributed to the asymmetric stretching modes of the CH_3_ group (not shown in Figure 2a).

The presence of graphite fillers does not affect the position and relative intensities of the characteristic PLA bands. However, due to the presence of the delocalized π-electrons that affect the optical properties of graphite, there is a strong correlation between the size of the graphite grains and the intensity of the absorption background [16,17]. When the size of the graphite grains is comparable with the wavelength of IR radiation (4–20 µm), the strong absorption background from the graphite fillers partially obscures the characteristic bands from the PLA matrix (spectra MG394/PLA, MG1596/PLA, MG3096/PLA in Figure 2a).

In Raman spectra shown in Figure 2b, a band at 873 cm^−1^ attributed to the stretching modes of the C-COO group of PLA is seen. The following oscillation modes of PLA visible in FTIR spectra are also Raman-active: the stretching modes of the C-CH_3_ group appear at 1043 cm^−1^, symmetric stretching modes of C-O-C group is found at 1095 cm^−1^, the rocking modes of the CH_3_ group appears at 1126 cm^−1^, the asymmetric bending modes of the CH_3_ group appears at 1453 cm^−1^, the stretching modes of the ester group appear at 1769 cm^−1^. The strongest bands derived from PLA occur at 2881, 2946, and 3002 cm^−1^ and are attributed to the stretching of the CH and asymmetric stretching of the CH_3_ group.

The bands of the graphite filler are clearly visible in the Raman spectra. The disorder induced graphitic D band appears at about 1355 cm^−1^. Its intensity is relatively low compared to the main graphitic band that appears at 1580 cm^−1^. That indicates a well-ordered graphite structure and low concentration of the defects [18]. At 2730 cm^−1^, there is a 2D graphite band. It is red-shifted by 10 cm^−1^ in fillers with smaller grain sizes (MG192, MG394, MG1596 and MG3096). The intensity of the graphite bands in relation to the PLA bands is greater with increasingly smaller grains of the filler. This is indirect evidence of the homogeneity of the composite.

### 3.3. Thermal Behavior and Stability Analysis

**Figure 3:**
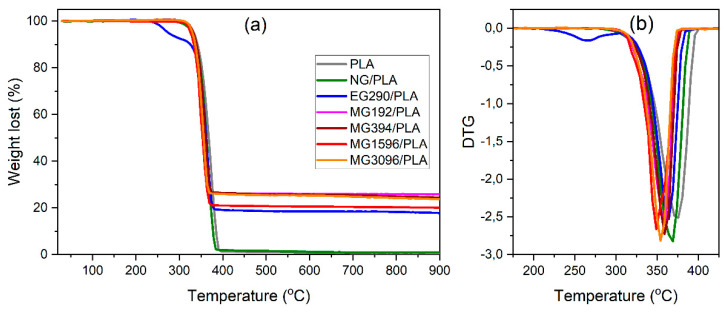
Thermogravimetric curves of graphite/PLA composites: (**a**) TG plots (**b**) DTG plots.

The thermal stability of the graphite/PLA composites was examined using TG analyses (Figure 3a). The temperature at which 5% weight loss occurred, *T*_5_, was assumed as the onset of the decomposition process. The maximum slopes of the TGA curves corresponding to the temperature at maximum degradation process rate, *T*_max_, were determined from the position of the peaks on the DTG curves shown in Figure 3b [19]. The thermal parameters of the composite samples, such as *T*_5_, *T*_max_, and mass loss at 900 °C, Δ*m*_900_, are summarized in Table 2.

The mass loss, Δ*m*_900_, at 900 °C roughly corresponds to the PLA content in the samples. Residues in the samples PLA and NG/PLA (0.6% and 1.0%, respectively) are related to the presence of impurities and ash formed as a result of thermal degradation of PLA. In the EG290/PLA sample, weight loss is observed when the temperature exceeds 200 °C. This behavior indicates the expansion of expandable graphite EG290 in an inert atmosphere. The TG analyses in air (not presented in this paper) show that, at temperatures between 200 and 300 °C, the EG290 loses approximately 75% of its mass. The weight loss in an inert gas atmosphere is much smaller (10%), however it occurs at the same temperatures. In the case of other composites, the residues at 900 °C correspond to the content of graphite micropowders in the materials.

The drop in the *T*_5_ and *T*_max_ of the crude PLA after the melt mixing (sample NG/PLA) is apparent, indicating that PLA undergoes thermal degradation upon processing. As shown by Signori et al. [20], during melt mixing, polymer chain scission and recombination occurs, which result in decreasing average number molecular weight and increasing polydispersity index. As a consequence, the thermal stability of the resin decreases. A further drop in the onset decomposition temperature, *T*_5_, and maximum process rate, *T*_max_, is observed in the samples containing graphite fillers. This result is inconsistent with previous observations reported for expandable graphite in [19] and graphite nanoplatelets [21]. It has been suggested that graphite filler has no major influence on the course of PLA decomposition under nitrogen atmosphere and only weak interaction occurs between PLA and its volatile decomposition products [21]. The slight decrease in the decomposition temperature of PLA is associated with the high thermal conductivity of graphite and graphene [22]. The presence of graphite filler facilitates heat transfer in the sample volume [23,24,25], thereby reducing the thermal decomposition temperature. High thermal conductivity begins to play a significant role at high graphite filler concentrations. This may be another reason for the discrepancy between the obtained results and previously reported observations. The process is the more efficient the more homogeneous the composite structure is. The homogeneity of the structure is influenced by the grain size of the filler. In fact, we observe a tendency for the thermal decomposition temperature to drop as the size of graphite grains decreases.

**Figure 4:**
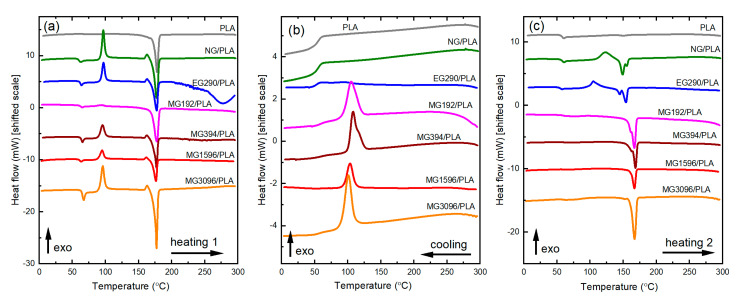
DSC analyses of graphite/PLA composites: (**a**) first heating, (**b**) cooling, (**c**) second heating.

To investigate more deeply the effect of melt mixing in the presence of different graphite fillers on the stability of the PLA matrix, the DSC traces of graphite/PLA composites were compared with pristine materials (Figure 4). Thermal data, such as the glass transition temperature (*T*_g_), crystallization temperature (*T*_c_), cold crystallization temperature (*T*_cc_), melting temperature (*T*_m_), crystallization enthalpy (Δ*H_c_*), cold crystallization enthalpy (Δ*H_cc_*), and melting enthalpy (Δ*H_m_*), are summarized in the Table 3.

The first heating scan of the crude PLA pellets showed an endothermic peak corresponding to the melting of the polymer ($T_{m}=177.8\;{}^{\circ}C$). This peak is not detectable in the second heating scan, proving that the slow crystallization rate of high molecular weight PLA is not conducive to the development of crystalline domains upon cooling [20]. Interestingly, the first heating scan of the processed PLA (NG/PLA) showed an endothermic glass transition at 60.3 °C, followed by an exothermic peak at 96.8 °C (cold crystallization) and an endothermic peak at 177.5 °C (melting). The cold crystallization peak is related to the reorganization of amorphous domains into crystalline ones caused by increased macromolecular mobility and flexibility of the processed polymer. Graphite fillers affect the position of the exothermic peak which is slightly shifted to lower temperatures. As revealed by molecular simulations, local mobility of the polymer chains near the graphite phase are highly anisotropic and dramatically reduced in the direction perpendicular to the graphite basal planes [26]. Thus, graphite filler can act as a nucleating agent that promotes the crystallization process. As a consequence, the *T*_cc_ decreases. The effect of the *T*_cc_ lowering does not occur in the composite containing expandable graphite (sample EG290/PLA) due to the large size of graphite flakes (~400 μm) which makes it difficult to obtain good dispersion of graphite in the polymer matrix. Graphite fillers affect the cold crystallization enthalpy, Δ*H_cc_*, as well. In general, the enthalpy change associated with cold crystallization is lower in the presence of graphite filler. The observed differences can be explained by the quality of the crystallites formed during cold crystallization. The lower crystallization enthalpy results in the formation of less perfect crystallites, capable of recrystallization during DSC analysis. The melting point, *T*_m_, of all samples is comparable. The melting enthalpy, Δ*H_m_*, of the processed NG/PLA sample is similar to the crude PLA and about 15–20 J/g higher than the melting enthalpy of the graphite containing samples. Since the crystallinity estimated from the first heating scan is at a similar level in the melt mixed PLA and melt mixed graphite/PLA composites (except for MG192/PLA sample), it can be assumed that the quality of the formed crystallites is responsible for the change of the melting enthalpy.

It is worth noting that the first DSC scan of the composite EG290/PLA showed a broad endothermic peak above 200 °C corresponding to the thermal expansion of the expandable graphite EG290.

Cooling scans confirmed the promoting role of the graphite filler in the crystallization process [27]. The exothermic crystallization peaks appeared only in the samples containing graphite filler. The exception is expandable graphite (EG290 filler), which is graphite chemically treated in concentrated sulfuric acid. As the cooling curves of PLA, NG/PLA, and EG290/PLA samples are similar to each other, we conclude that sulfur compounds released during the first heating inhibit the crystallization of the PLA matrix.

During the second heating, bimodal thermal transitions occur, which are manifested by two endothermic peaks. The two melting peaks are related to the presence of two types of crystallites of different sizes and disorder [28]. The melting peak at lower temperature is attributed to the melting and recrystallization of the primary crystals into a more stable form. The second peak appearing at higher temperature corresponds to the melting of the newly formed crystals [29]. The shift of the position of both melting peaks towards higher temperatures is very pronounced in composites with unmodified graphite. This supports the occurrence of less degradation of the polymer matrix during the first heating and the nucleation action of graphite filler. The melting point increase is not observed in the composite containing expanded graphite. Probably, the sulfur compounds released during the first heating accelerate the degradation of the polymer matrix.

The room temperature crystallinity, *X_c_*, in the neat polymer decreased after each heating cycle. The *X_c_* of the pristine PLA estimated from the first heating curve was found to be 62%. In PLA subjected to the melt mixing (sample NG/PLA), the *X_c_* decreased to the 29%. The second heating caused the crystallinity to drop to almost zero in both the PLA and NG/PLA samples. The presence of graphite admixture causes a slight decrease in crystallinity observed during the first heating cycle. However, the role of chemically unmodified graphite as a nucleating agent is growing with the history of processing. It is manifested by increased crystallinity estimated from the second heating scan. The highest percent crystalline content (51%) was found in the composite containing graphite with a grain size of 14 µm (sample MG394/PLA). In the remaining samples with unmodified graphite as filler, the crystallinity is slightly lower, but still exceeds 40%. This behavior is evidence of the nucleating action of the graphite filler. On the other hand, the use of the expandable graphite as a filler (EG290) does not produce this effect. Crystallinity determined from the second heating data increased slightly in relation to neat PLA to the level of 6%. Expandable graphite is intercalated with sulfur compounds that are released during thermal treatment and inhibit the crystallization processes.

It is known that graphite fillers may affect the thermal stability of biodegradable polyesters such as PLA, which usually inhibit thermal oxidation [30].

**Figure 5:**
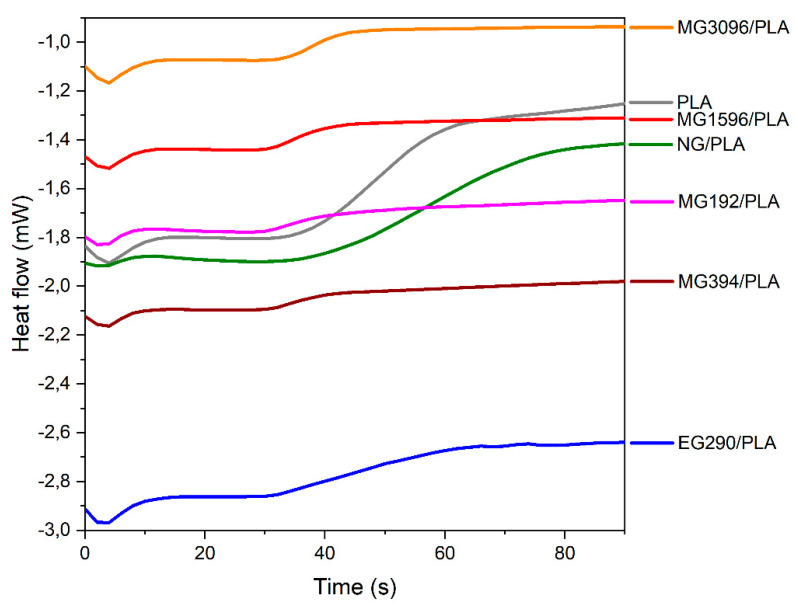
DSC thermograms at 230 °C for oxidation induction time analyses.

To investigate how the melt mixing and presence of graphite fillers affect the resistance to oxidation, we have recorded isothermal thermograms at 230 °C (Figure 5). The changes in heat flow accompanying oxidation are greater in PLA samples than in graphite/PLA composites.

The onset times of the exothermic reaction (OIT) are summarized in Table 4. No differences between PLA and NG/PLA were observed, proving that processing of the neat PLA does not affect the resistivity of PLA to oxidation. The thermal stability of graphite/PLA composites is reduced compared to neat PLA. The reduced value of OIT is mainly caused by the porous morphology of composite samples (higher specific surface) that facilitates oxygen access to the PLA matrix.

The percent crystalline content of a polymer can be used to tailor the melt flow rate for specific melt processing. Table 5 summarizes the MFR values of the PLA and graphite/PLA composites obtained by melt mixing at 190 °C. The MFR of the PLA subjected to the melt mixing is slightly lower than that of the crude PLA, indicating a correlation between the changes in the polymer structure resulting from the melt processing and the flow resistance [31]. However, confirmation of this observation requires further work involving the measurement of the intrinsic viscosity of polymer solutions. The MFR values of graphite/PLA composites are about half lower than the neat PLA. Many authors attribute the decrease in MFR to increased flow resistance associated with the use of solid filler [32,33]. As reported by Wang et al. [34], collisions and friction between the filler particles limit the mobility of the molecular chains. A similar explanation for the decrease in MFR values in the polymer composites with graphite nanoplatelets has been proposed by Duguay et al. [35], who also have linked the flow resistance with the grain size of the nanoplatelets. Indeed, in the MG394/PLA, MG1596/PLA, and MG3096/PLA samples containing, respectively, 14, 10, and 6 µm graphite grains, we also observe an increase in the MFR value with decreasing filler grain size.

On the other hand, the MFR values correlate with the percent crystalline content of the PLA matrix derived from the DSC second heating scan shown in Table 3. It is worth noting that the crystallinity estimated from the DSC second heating scan accurately reflects the processing history of materials subjected to MFR tests. Comparing the DSC results with the MFR values, we come to the conclusion that the increased crystallinity of the polymer matrix has a direct impact on the decreased MFR value. The greater the percent of the crystalline content, the lower the melt flow rate. Even with a relatively high content of graphite filler (~20%), the crystallinity of the polymer matrix has a greater influence on the flow rate than the friction between the grains of the graphite filler.

The extremely high MFR value of the EG290/PLA composite is related to the low crystallinity of the polymer matrix and delamination processes of expandable graphite at elevated temperatures, which radically reduce the shear friction in the melted composite.

## 4. Conclusions

The introduction of graphite fillers into the PLA matrix resulted in the modification of the thermal and melt flow properties of the composites. FTIR and Raman spectroscopy analyses do not indicate changes in the chemical structure of the polymer matrix and the graphite filler.

TG analyses of the materials under nitrogen show a slight drop of the thermal decomposition temperature of the PLA matrix mixed with graphite. We attribute this behavior to the high thermal conductivity of graphite that facilitates heat transfer in the sample volume, thereby reducing the thermal decomposition temperature. The DSC data track the thermal transitions that occur during the thermal processing. While no effect of graphite filler on the melting temperature was observed during the first heating scan, an apparent increase in the melting temperature is seen during the second heating, proving the slower degradation of the PLA matrix. The influence of the graphite filler on the crystallinity of the polymer matrix is also enhanced with the thermal processing history. The crystallinity determined from the first heating scan is slightly lower in the presence of graphite. The value of this parameter obtained from the second heating increases from 0% in the neat polymer to about 50% in graphite/PLA composites. This result is prospective for the design of cheap, biodegradable composite materials for a specific melt processing (melt molding, 3D printing, etc.).

The addition of graphite to PLA causes a slight reduction in the oxidation induction time. Lower resistance to oxygen is related to the porosity of the composite after melt mixing. MFR values are strictly correlated with the crystallinity of the polymer matrix estimated from the DSC second heating data. The greater the percent of the crystalline content, the lower the melt flow rate. Hence, we conclude that the presence of the graphite filler has a secondary effect on the melt flow rate of the composites. This statement does not apply to the expandable graphene filler EG290 (which is indeed an intercalated graphite compound). Delamination of graphite layers that occurs at elevated temperature reduces the shear friction that results in the extremely high melt flow rate We show that the correlation between the size of the graphite filler grains and thermal processing parameters of the graphite/PLA composites is rather weak. Taking into account the melting point and crystallinity, the optimal size of the graphite grains in terms of the composite processability is ~14 µm. Nevertheless, the results presented show the path to designing composite materials based on graphite of industrial grade for specific thermal processing.

## Figures and Tables

**Table 1:** Labeling, composition and processing method of the tested graphite/PLA composites.

Code	PLA (wt%)	Graphite (wt%)	Sample Preparation Method
PLA	PLA3260HP (100)	-	Unprocessed granulate
NG/PLA	PLA3260HP (100)	-	Melt mixing
EG290/PLA	PLA3260HP (73)	EG290 (27)	Melt mixing
MG192/PLA	PLA3260HP 74	MG192 (26)	Melt mixing
MG394/PLA	PLA3260HP (75)	MG394 (25)	Melt mixing
MG1596/PLA	PLA3260HP (79)	MG1596 (21)	Melt mixing
MG3096/PLA	PLA3260HP (76)	MG3096 (24)	Melt mixing

**Table 2:** Thermal parameters obtained from TG and DTG curves of graphite/PLA composites.

Sample	*T*_5_ (°C)	*T*_max_ (°C)	Δ*m*_900_ (%)
PLA	333	376	99.4
NG/PLA	329	369	99.0
EG290/PLA *	-	364	82.2
MG192/PLA	328	360	74.2
MG394/PLA	332	364	75.7
MG1596/PLA	328	352	79.9
MG3096/PLA	327	359	76.3

* Due to the overlapping weight loss associated with EG290 expansion and PLA thermal degradation, *T*_5_ is difficult to quantify.

**Table 3:** Thermal data obtained by DSC for graphite/PLA composites.

Sample	Heating 1						Cooling		Heating 2			
	*T*_m_ [°C]	Δ*H_m_* [J/g]	*T*_g_ [°C]	*T*_cc_ [°C]	Δ*H_cc_* [J/g]	*X_c_*^1^ [%]	*T*_c_ [°C]	Δ*H_c_* [J/g]	*T*_m_ [°C]	Δ*H_m_* [J/g]	*T*_g_ [°C]	*X_c_*^2^ [%]
PLA	177.78	57.22	-	-	-	62	-	-	-	-	57.56	0
NG/PLA	177.45	59.72	60.29	96.84	32.31	29	-	-	149.20 155.25	21.98	57.91	3
EG290/PLA	177.58	40.54	63.97	97.13	21.74	27	-	-	144.64 154.09	2.48 11.06	57.50	6
MG192/PLA	177.92	43.10	65.11	94.28	1.82	59	105.14	23.49	162.47 166.86	32.06	-	46
MG394/PLA	177.35	43.66	65.45	95.56	22.18	31	107.96	28.53	163.06 168.44	35.71	-	51
MG1596/PLA	176.33	45.02	60.69	94.90	21.75	31	103.41	22.14	166.88	31.68	-	43
MG3096/PLA	176.66	36.05	65.95	96.13	22.88	30	105.30	26.86	162.40 168.07	34.09	-	49

**Table 4:** OIT values with standard deviations.

Sample	OIT [s]
PLA	38 ± 2
NG/PLA	38 ± 3
EG290/PLA	32 ± 2
MG192/PLA	31 ± 1
MG394/PLA	31 ± 2
MG1596/PLA	31 ± 1
MG3096/PLA	33 ± 4

**Table 5:** MFR values with standard deviations.

Sample	MFR (g/10 min)
PLA	69.1 ± 3.5
NG/PLA	64.9 ± 1.6
EG290/PLA	>125
MG192/PLA	31.3 ± 1.2
MG394/PLA	23.9 ± 0.9
MG1596/PLA	25.9 ± 1.7
MG3096/PLA	31.4 ± 1.2

## Data Availability

The data presented in this study are available on request from the corresponding author.
